# Dual function of peroxiredoxin I in lipopolysaccharide-induced osteoblast apoptosis via reactive oxygen species and the apoptosis signal-regulating kinase 1 signaling pathway

**DOI:** 10.1038/s41420-018-0050-9

**Published:** 2018-04-27

**Authors:** Hao Feng, Ziyu Li, Juan Du, Jing Sun, Wei Feng, Dongfang Li, Shanshan Liu, Wei Wang, Hongrui Liu, Norio Amizuka, Minqi Li

**Affiliations:** 1 Department of Bone Metabolism, School of Stomatology Shandong University, Shandong Provincial Key Laboratory of Oral Tissue Regeneration, Jinan, China; 2 Department of Endodontics, Jinan Stomatological Hospital, Jinan, China; 3 Department of Developmental Biology of Hard Tissue, Graduate School of Dental Medicine, Hokkaido University, Sapporo, Japan

## Abstract

Lipopolysaccharide (LPS)-induced osteoblast apoptosis is a prominent factor to the defect in periodontal tissue repair in periodontal disease. LPS challenge contributes to the production of reactive oxygen species (ROS) in periodontitis, and peroxiredoxin 1 (Prx1) is an antioxidant protein that protect cells against oxidative damage from ROS. Without LPS stimulation, apoptotic rates were higher in both Prx1 knockout (Prx1^KO^) and Prx1 overexpression (Prx1^OE^) cells compared with wild type. After LPS stimulation, intracellular ROS in Prx1^KO^ cells showed the highest level and Prx1^OE^ cells showed the least. Treatment with LPS significantly elevated the expression of Bax, Cyto-c, and caspase 3 in Prx1^KO^ cells compared with wild type, although this could be completely abolished by NAC. In Prx1^OE^ cells, the expression and activation of ASK1 were significantly increased, and this was slightly reduced by LPS stimulation. NQDI-1 completely abolished the increased phosphorylation of JNK and p38 and the expression of caspase 3 in LPS-stimulated cells. These results indicate that Prx1 eliminates intracellular ROS and exhibits a cytoprotective role in LPS-induced apoptosis. However, under physiological conditions, Prx1 overexpression acts as a H_2_O_2_ messenger, triggering the expression of ASK1 and its downstream cascades.

## Introduction

Bone remodeling involves the restructuring of existing bone, which undergoes constant resorption and formation. Excessive bone resorption is the cause of bone loss observed in periodontitis, which is a chronic microbial infectious disease resulting in destruction of the periodontium. It is characterized by tissue inflammation, alveolar bone resorption, and eventually tooth loss^1,2^. The increased apoptosis of osteoblasts and osteocytes is one of the mechanisms that underlie the reduced bone formation and fragility that characterize periodontitis-induced bone destruction^3^. Thus, understanding the mechanisms of apoptosis in osteoblasts is imperative to developing therapeutic strategies.

Lipopolysaccharides (LPS), an important component of the outer membrane of Gram-negative bacteria, are involved in the pathogenesis of periodontal diseases and play an important role in alveolar bone resorption^4–6^. Accumulated data have shown that LPS directly induce apoptosis in many cell types, including macrophages, vascular endothelial cells, and hepatocytes^7–9^. LPS-induced apoptosis in osteoblasts and periodontal ligament fibroblasts is seen as an important contributing factor leading to defects in periodontal tissue repair in periodontal and periapical diseases^10^, but the mechanism of LPS-induced apoptosis in these cells remains unclear.

Following LPS recognition, Toll-like receptor undergoes oligomerization and recruits its downstream adapters to activate other molecules within the cell, leading to induction of the inflammatory response, and the subsequent production of reactive oxygen species (ROS)^11^. ROS acts as a second messenger in signal transduction and gene regulation in a variety of cell types under several biological conditions such as cell growth, differentiation, progression, and death^12,13^. It can directly trigger apoptosis by causing excessive protein, lipid, and nucleic acid oxidation, but also has the potential to regulate pathways involving Bcl-2 family members and caspases^14^. Oxidation of the anionic phospholipid cardiolipin in the inner mitochondrial membrane is also proposed to play an important role in mitochondrial disruption and cytochrome *c* (Cyto-c) expression, which occurs during apoptosis^15^. Recent experimental evidence has shown that LPS-induced proinflammation and cell death are highly dependent on ROS and related signaling pathways^16–18^. Thus, ROS has a critical role in LPS-induced osteoblast apoptosis.

Peroxiredoxins (Prxs) belong to an important superfamily of small non-seleno peroxidases that scavenge hydrogen peroxide (H_2_O_2_) and organic hydroperoxide, and are essential for maintaining intracellular ROS homeostasis^19^. On the basis of the numbers of conserved cysteine (Cys) residues participating in the redox reaction, Prxs are divided into typical 2-Cys Prxs (including Prx1−6), atypical 2-Cys Prx (Prx5), and 1-Cys (Prx6)^20^. Prx1 is one of the typical 2-Cys Prxs, and is involved in multiple physiological and pathological processes, including proliferation, apoptosis, inflammation, and cancer^21–23^. In particular, it has been shown that Prx1 suppresses oxidative stress-induced cell apoptosis through direct or indirect interactions with a variety of apoptosis-regulating kinases and enzymes such as apoptosis signal-regulating kinase 1 (ASK1) and p38 in a cell type- and stimulus-dependent manner^24^. Moreover, Prx1 overexpression was shown to inhibit the activation of ASK1, resulting in the inhibition of downstream signaling cascades such as c-Jun N-terminal kinase (JNK) and the p38 pathway^25^. In Prx1 knockdown cells, the sensitivity of ROS was strongly increased, and ASK1, P38, and JNK were rapidly activated, leading to apoptosis in response to H_2_O_2_^26,27^. Thus, Prx1 functions as a protective factor in cell death, but its role in LPS-induced osteoblast apoptosis remains unclear.

Because Prxs have recently been identified as essential negative regulators of LPS-induced inflammatory and apoptosis^28,29^, we investigated whether Prx1 regulates LPS-induced osteoblast apoptosis. In this study, we demonstrated that Prx1 acts as either an anti-apoptotic or a pro-apoptotic factor depending on its intracellular level, through modulating ROS or ASK1 signaling, respectively.

## Results

### LPS affects osteoblast proliferation and apoptosis in a dose- and time-dependent manner

**Fig. 1:**
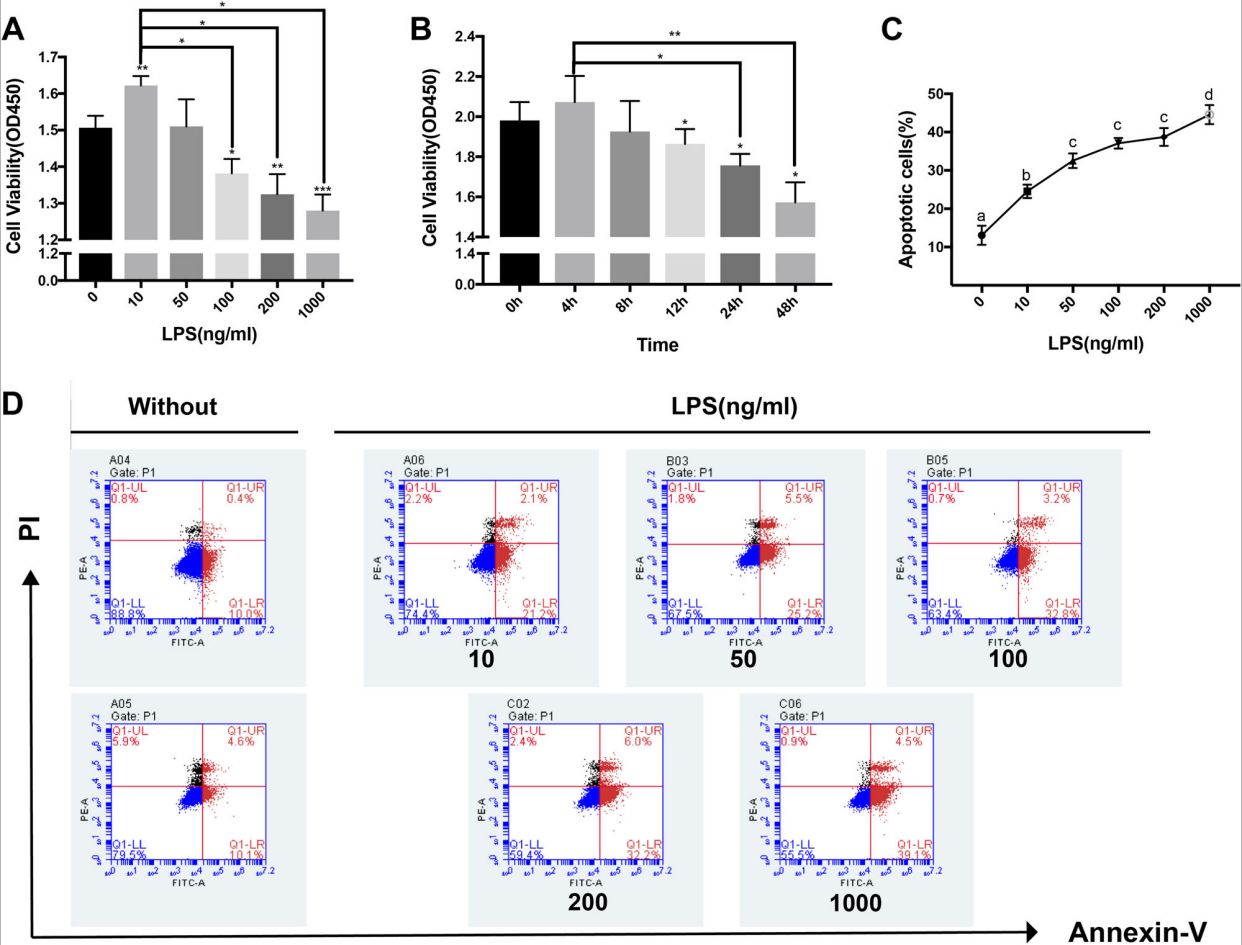
LPS causes the reduction of cell viability in dose- and time-dependent manners and increases the number of apoptotic cells. **a** MC3T3-E1 cells were treated with LPS at different concentrations for 24 h. Cell viability was evaluated using CCK8 assays. **b** MC3T3-E1 cells were treated with 100 ng/ml LPS for the indicated time. **c**,** d** MC3T3-E1 cells were treated with LPS at different concentrations for 24 h and then stained with PI and Annexin V, and the percentage of apoptotic cells was analyzed by flow cytometry assays. *P < 0.05, **P < 0.01. All values represent the mean ± SD from three independent experiments. Different letters indicate significant differences between groups (*P* < 0.05)

To determine the effect of LPS on cell viability in osteoblasts, MC3T3-E1 cells were treated with different concentrations of LPS for 24 h or with 100 ng/ml LPS for various times. CCK8 assays showed that cell viability in osteoblasts exposed to 100, 200, or 1000 ng/ml LPS was significantly reduced compared with non-treated cultures in a dose-dependent manner (Fig. 1a). After treatment with 100 ng/ml LPS, a significant reduction in cell viability was observed at 12 h, 24 h, or 48 h compared with the control group (Fig. 1b). Flow cytometry analysis of the apoptotic rate of osteoblasts following treatment with different concentrations of LPS for 24 h showed dose-dependent increases in apoptosis compared with the control group (Fig. 1c, d).

### Prx1 expression is induced by LPS via intracellular ROS in osteoblasts

**Fig. 2:**
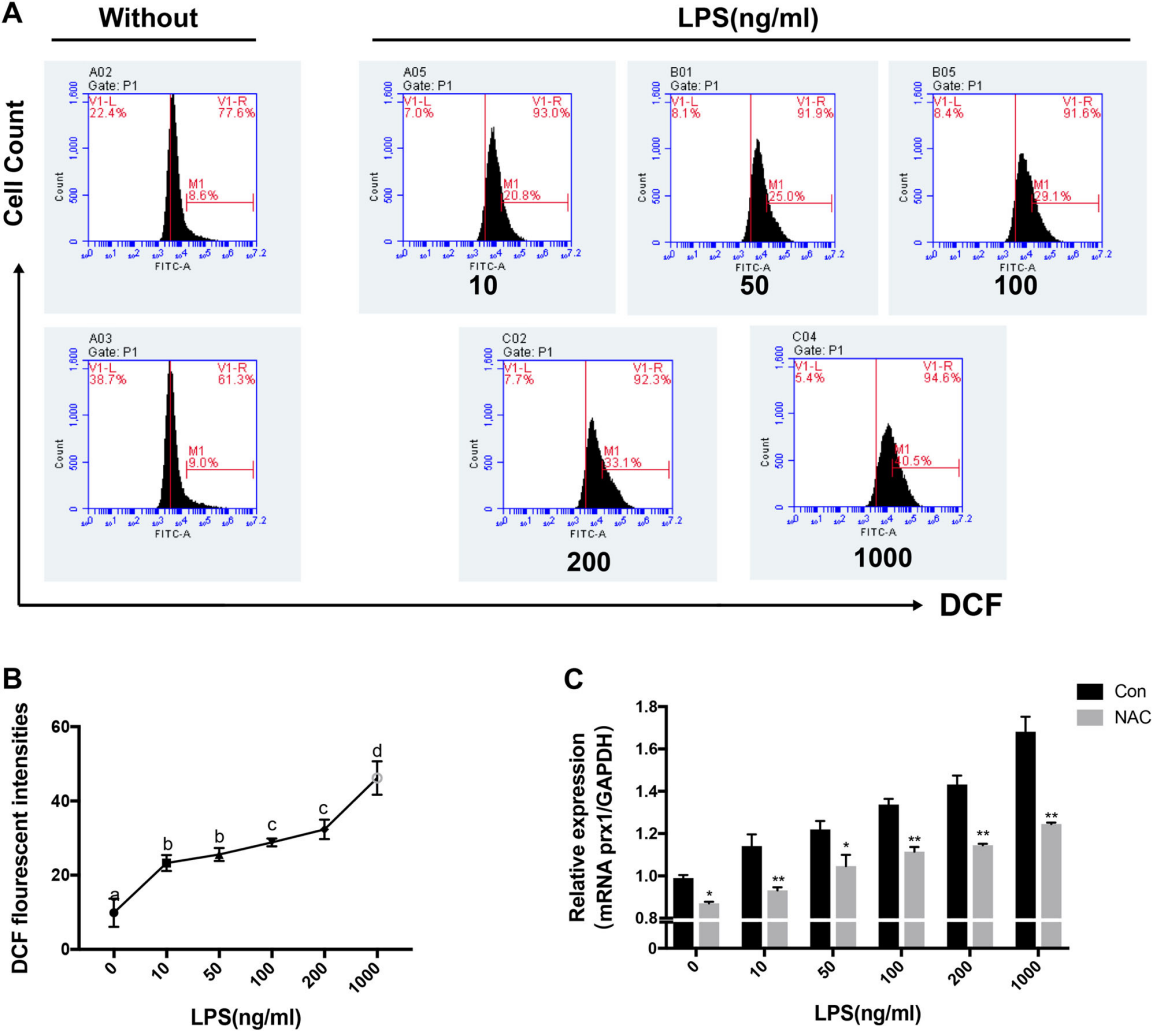
LPS increases the level of ROS and the expression of Prx1 in MC3T3-E1 cells. **a**,** b** MC3T3-E1 cells were treated with different concentrations of LPS for 24 h, and the level of ROS was analyzed by flow cytometry assays. **c** MC3T3-E1 cells were treated with the indicated concentrations of LPS for 24 h after being pretreated for 3 h in the absence or presence of NAC, and the expression of Prx1 gene was assessed by qRT-PCR. *P < 0.05, **P < 0.01. The results are expressed as mean ± SD from three independent experiments. Means with different letters differ significantly from each other (*P* < 0.05)

To investigate whether ROS is increased by LPS stimulation in osteoblasts, MC3T3-E1 cells were treated with different concentrations of LPS, and ROS levels analyzed by flow cytometry after incubation with 2′,7′-dichlorodihydrofluorescein diacetate (H2DCFDA). The level of intracellular ROS was significantly increased by LPS stimulation in a dose-dependent manner (Fig. 2a–c). *N*-acetyl-cysteine (NAC) was then applied to verify the role of ROS in LPS-induced Prx1 overexpression, and *Prx1* mRNA transcription levels were determined by quantitative real-time PCR. After treatment with LPS for 24 h, *Prx1* mRNA transcription was significantly increased; however, it was markedly attenuated by the addition of NAC.

### Establishment of Prx1 knockout and overexpressing MC3T3 cell lines

**Fig. 3:**
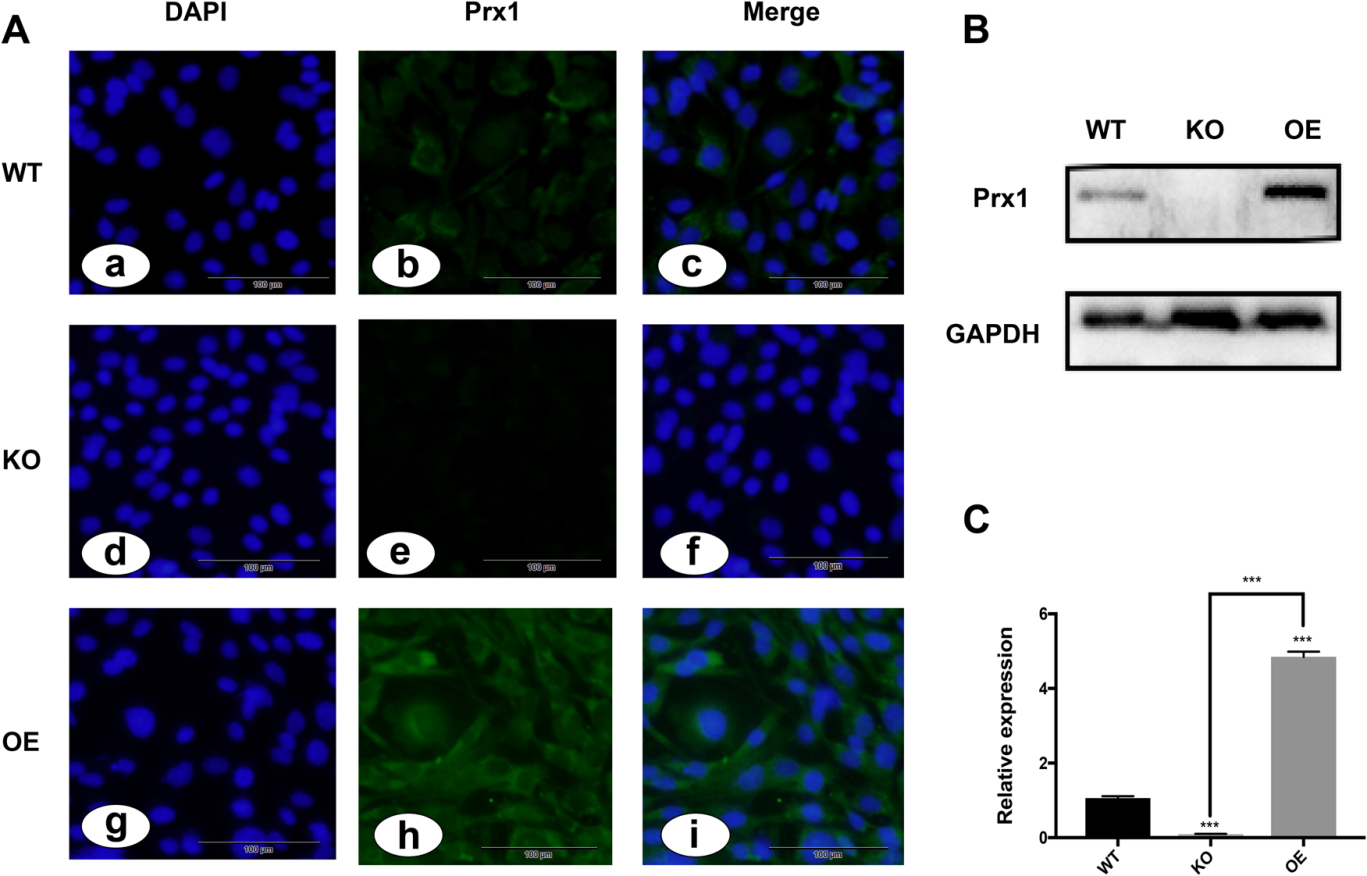
The expression of Prx1 in Prx1^KO^, Prx1^OE^ and wild-type MC3T3-E1 cells. **a**(a–c) Wild-type MC3T3-E1 cells of DAPI staining (a), expression of Prx1(b), and merge of (a, b) (c). d–f Prx1^KO^ cells of DAPI staining (d), expression of Prx1 (e), and merge of (d, e) (f). g–i Prx1^OE^ cells of DAPI staining (g), expression of Prx1 (h), and merge of (g, h) (i). **b** Western blot analysis was used to detect Prx1 proteins in wild-type, Prx1^KO^ and Prx1^OE^ cells. **c** the relative expression of Prx1 mRNA in wild-type, Prx1^KO^ and Prx1^OE^ cells was measured by qRT-PCR. ***P < 0.005

To evaluate the function of Prx1 in LPS-induced apoptosis, *Prx1* knockout (Prx1^KO^) and *Prx1* overexpression (Prx1^OE^) MC3T3-E1 cells were generated by the CRISPR/Cas9 system and Integration Operating System, respectively. Cellular immunofluorescence and western blot assays showed that Prx1^KO^ cells had significantly decreased Prx1 protein levels, while Prx1^OE^ cells had dramatically increased Prx1 protein levels compared with wild-type cells (Fig. 3a, b). Quantitative real-time PCR also revealed that *Prx1* mRNA levels were reduced in Prx1^KO^ cells, while their expression in Prx1^OE^ cells was five times higher than in wild-type cells (Fig. 3c).

### Prx1 affects LPS-induced apoptosis by regulating intracellular ROS

**Fig. 4:**
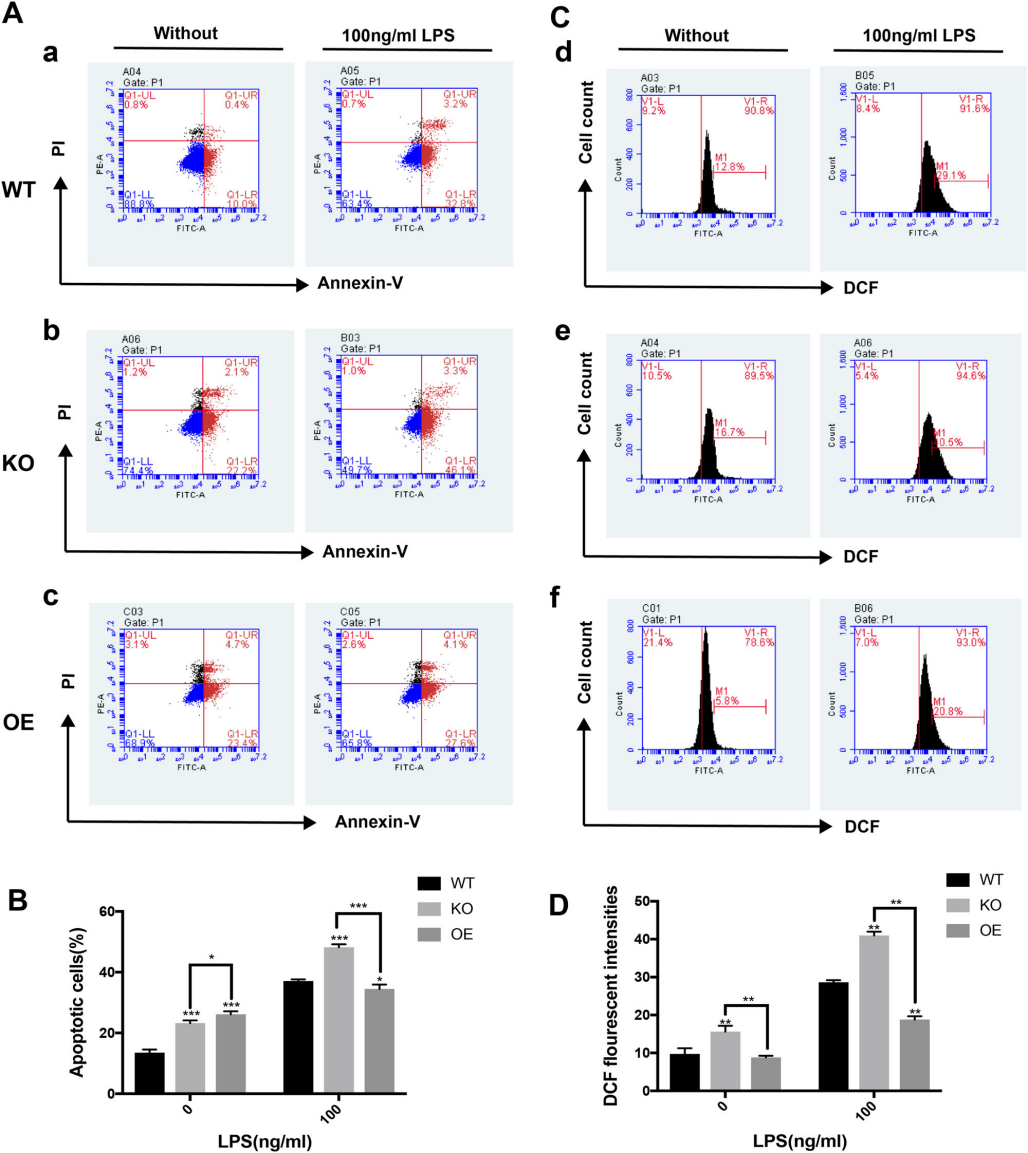
Prx1 controls the level of intracellular ROS during LPS-induced apoptosis. **a**, **b** Wild-type cells (a), Prx1^KO^ cells (b), and Prx1^OE^ cells (c) were treated with the absence or presence of 100 ng/ml LPS, and the amount of apoptotic cells was assessed by flow cytometry assays. **c**, **d** Wild-type cells (d), Prx1^KO^ cells (e), and Prx1^OE^ cells (f) were treated with or without 100 ng/ml LPS for 24 h, and after incubation with H2DCFDA, the level of ROS was analyzed by flow cytometry. *P < 0.05, **P < 0.01, and ***P < 0.005. All values represent the mean ± SD from three independent experiments

To further investigate whether Prx1 affects LPS-induced apoptosis by regulating intracellular ROS, cells were exposed to 100 ng/ml LPS for 24 h. They were then stained with Annexin V, and the level of apoptosis was assessed by flow cytometry (Fig. 4a). In the absence of LPS stimulation, the number of apoptotic cells in both Prx1^KO^ and Prx1^OE^ cells was unexpectedly higher than in wild-type cells, while no obvious difference in the number of Annexin V^+^ cells was observed between Prx1^KO^ cells and Prx1^OE^ cells. However, after treatment with LPS, the number of apoptotic Prx1^OE^ cells was markedly decreased compared with Prx1^KO^ cells and wild-type cells, while the number of apoptotic Prx1^KO^ cells was the highest of all groups (Fig. 4b).

Endogenous ROS was measured with the H2DCFDA probe using flow cytometry (Fig. 4c). In the absence of LPS stimulation, the DCF fluorescence intensity of Prx1^KO^ cells was extremely higher than both Prx1^OE^ cells and wild-type cells. However, when cells were exposed to 100 ng/ml LPS, the DCF fluorescence intensity in Prx1^OE^ cells was less than half that in Prx1^KO^ cells and significantly lower than in wild-type cells (Fig. 4d).

### The peroxidase activity of Prx1 suppresses LPS-induced apoptosis via inhibiting ROS

**Fig. 5:**
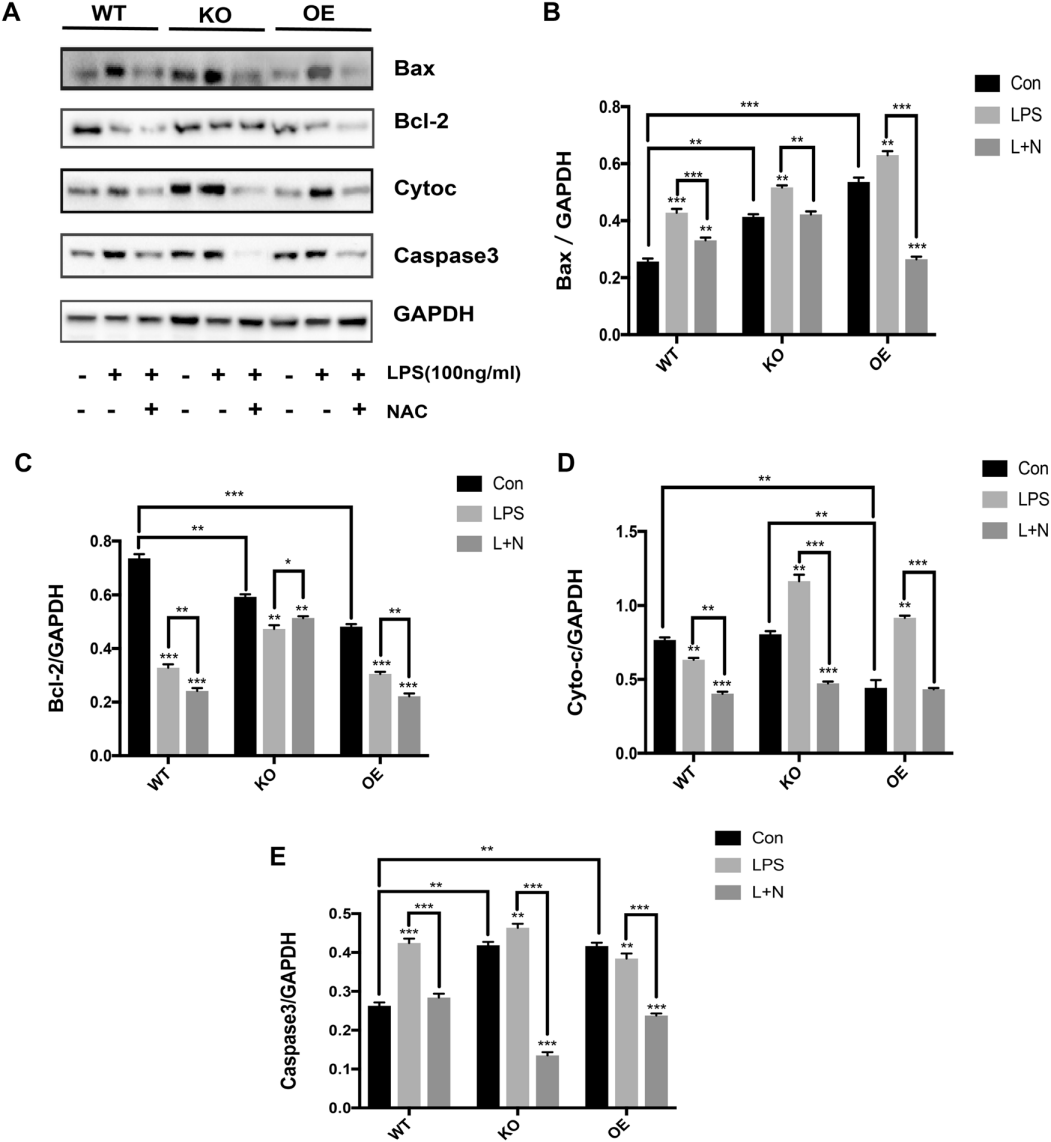
The peroxidase activity of Prx1 has a vital role in LPS-induced apoptosis by inhibiting ROS. **a** Wild-type, Prx1^KO^, and Prx1^OE^ cells were each divided into three groups, which were control groups, groups treated with 100 ng/ml LPS, and groups treated with 100 ng/ml LPS and NAC. Western blot analysis was then performed with antibodies against Bax, Bcl-2, Cyto-c, caspase 3, and GAPDH, and band intensity was measured with densitometry. **b**–**e** Data are representative of three independent experiments as the mean ± SD. *P < 0.05, **P < 0.01 and ***P < 0.005

Based on these findings, we next considered how the peroxidase activity of Prx1 was associated with LPS-induced apoptosis. Western blot assays were therefore used to assess whether the peroxidase activity of Prx1 could regulate ROS-mediated expression of the Bcl-2 family and caspase 3 in response to LPS. In the absence of LPS stimulation, the expression of Bax and caspase 3 in Prx1^KO^ and Prx1^OE^ cells was higher than in wild-type cells, which was contrary to the expression of Bcl-2 (Fig. 5a). However, after LPS stimulation, the expression of Bax and caspase 3 was increased while Bcl-2 was decreased in Prx1^OE^ and wild-type cells compared with control groups; these changes were abolished after the addition of NAC (Fig. 5b, c, e).

Next, the role of Prx1 in the expression of Cyto-c in response to LPS stimuli was examined (Fig. 5a). Prx1^KO^ cells showed an extremely high level of Cyto-c expression compared with the other cell types, both with or without LPS stimulation. This Cyto-c expression could be abolished by NAC (Fig. 5d).

### The overexpression of Prx1 promotes ASK1 activation by inhibiting the activity of thioredoxin towards ASK1

**Fig. 6:**
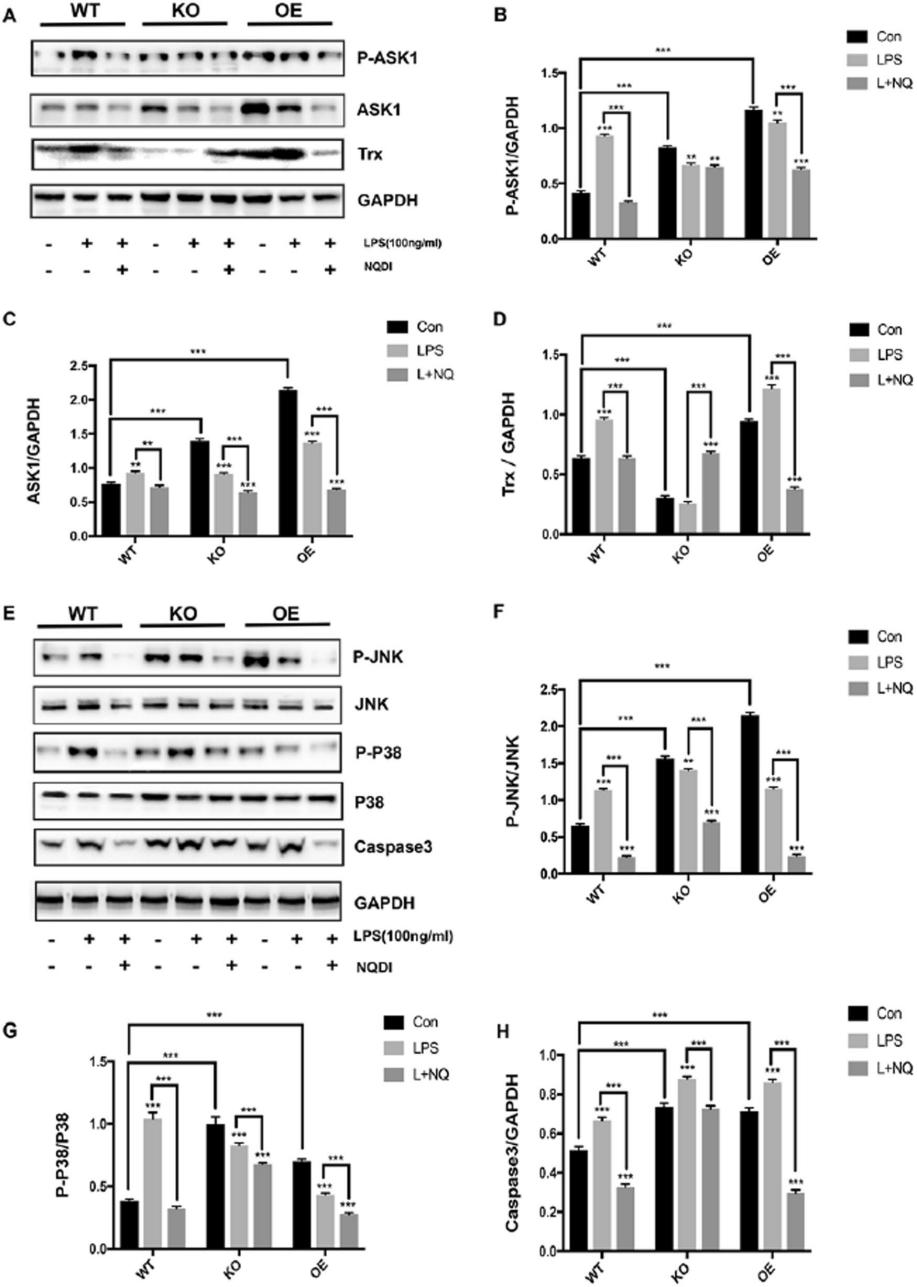
The overexpression of Prx1 promotes ASK1 activation by inhibiting the activity of Trx towards ASK1. **a**–**d** Wild-type, Prx1^KO^, and Prx1^OE^ cells were each divided into three groups, which were control groups, groups treated with 100 ng/ml LPS, and groups treated with 100 ng/ml LPS and NQDI. Western blot analysis was then performed with antibodies against P-ASK1, ASK1, Trx and GAPDH. **e**–**h** Wild-type, Prx1^KO^, and Prx1^OE^ cells were each treated with or without LPS or LPS with NQDI-1. The expression of P-JNK, JNK, P-P38, P38, caspase 3, and GAPDH were then measured using western blot analysis with corresponding antibodies. All values are the mean ± SD of three independent experiments. **P < 0.01 and ***P < 0.005

To investigate whether Prx1 overexpression is involved in other signal pathways in LPS-induced apoptosis, western blotting was used to measure increases in the ASK1-activated mitogen-activated protein kinase signal pathway in response to LPS stimuli (Fig. 6a, e). In the absence of LPS stimulation, the expression and activation of ASK1 in Prx1^OE^ cells were much higher than in Prx1^KO^ cells and wild-type cells. However, after LPS stimulation, Prx1^OE^ cells showed an attenuated activation and expression of ASK1, and wild-type cells showed a dramatic increase in its activation and expression (Fig. 6b, c). In the absence of LPS stimulation, downstream molecules such as JNK and p38 demonstrated a marked increase in expression in Prx1^OE^ cells after LPS stimulation; however, the expression of these downstream messengers was markedly decreased in Prx1^OE^ cells (Fig. 6f, g).

Trx expression was much higher in Prx1^OE^ cells than in Prx1^KO^ and wild-type cells without LPS stimulation (Fig. 6a). In response to LPS, no significant changes could be detected in the expression of Trx in Prx1^KO^ cells; in contrast, both Prx1^OE^ cells and wild-type cells presented with significantly elevated levels of Trx, with Prx1^OE^ cells showing the highest expression (Fig. 6d). LPS-induced apoptosis in Prx1^OE^ and wild-type cells was completely ablated by the addition of NQDI-1 (Fig. 6h), which has previously been shown to specifically inhibit the phosphorylation of ASK1 and ASK1-induced apoptosis^30^.

## Discussion

Alveolar bone loss, a hallmark of periodontitis progression, is mediated by host immune and inflammatory responses to microbial changes such as LPS^4^. During inflammatory responses, LPS significantly contributes to the production of ROS which acts as a second messenger and participates in a variety of pathological processes such as apoptosis^13,31^. Prxs are a group of thiol peroxidases that degrade H_2_O_2_ to eliminate intracellular ROS^32^. Although catalase and glutathione peroxidases have been considered the major enzymes responsible for protecting cells against oxidative stress, recent data on the reactivity and abundance of Prxs have revealed them to be prominent members of the antioxidant defenses and important players in cellular redox signaling^24,33^. Recently, LPS-induced apoptosis in osteoblasts and fibroblasts has been suggested to contribute to the defect in periodontal tissue repair in periodontal disease^10^. Hence, we investigated the role of Prx1 in LPS-induced osteoblast apoptosis.

LPS inhibits the expression of proteins such as alkaline phosphates, osteocalcin, and bone sialoprotein, which are dose- and time-dependently associated with osteoblast differentiation^34^. We showed that LPS inhibited cell proliferation in a dose- and time-dependent manner, and significantly increased the number of apoptotic cells. Furthermore, ROS production was augmented by LPS, which increased the expression of Prx1. This increased LPS-induced apoptosis from Prx1-deficient pre-osteoblasts appears to be linked to the antioxidant activity of Prx1, given that overall levels of ROS were significantly higher in Prx1-deficient cells after LPS stimulation than in wild-type cells. To further understand the function of Prx1 in ROS-induced apoptosis, we assessed the expression of Cyto-c which participates in electron transfer as part of the mitochondrial electron transport chain. Cyto-c generated by damaged mitochondria activates the initiator caspase 9 and its downstream caspases^15^. We observed much higher Cyto-c expression in Prx1^KO^ cells compared with other cells with or without LPS stimuli. These results indicate that the peroxidase activity of Prx1 provided protection against the harmful consequences of oxidative stress.

**Fig. 7:**
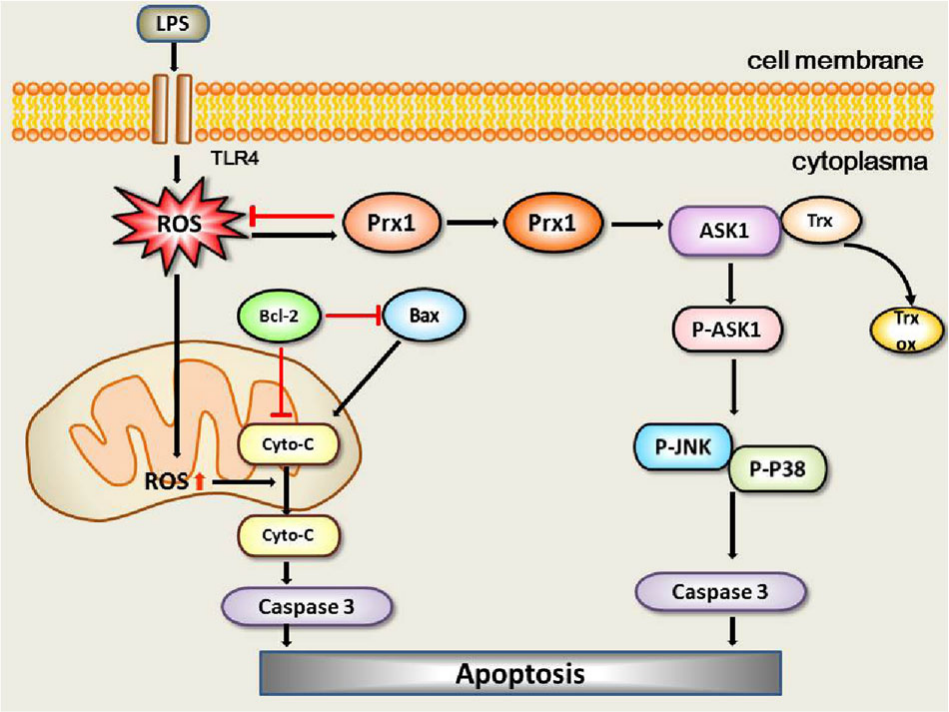
A possible model of cellular functions of bifunctional Prx1 modulating LPS-induced apoptosis via ROS and ASK1 signaling .

Lee and colleagues^25^ previously reported that Prx1 overexpression inhibited the activation of ASK1, resulting in the inhibition of downstream signaling cascades such as JNK and p38. ASK1 is a member of the mitogen-activated protein kinase kinase kinase family and is also involved in LPS-induced apoptosis^35^. Our current work suggests that Prx1 has dual functions in modulating the activation of ASK1, which are dependent on the peroxidase activity of Prx1 and Trx. We found that Prx1 overexpression significantly increased the expression and activation of ASK1 in the absence of LPS stimulation. This is in line with the previous finding that Prx1 acts as an H_2_O_2_ receptor, and transduces the signal to drive the oxidative activation of ASK1^36^. Moreover, in the present study, the levels of intracellular ROS and apoptosis in Prx1^OE^ cells were lower than in wild-type cells in response to LPS, indicating that the peroxidase activity of Prx1 may play a protective role in LPS-induced apoptosis. Trx has been shown to reduce the hyperoxidation of Prx1 and inhibit ASK1 activation by interacting with it under reducing conditions^37,38^. Here, we found that Trx was highly expressed in Prx1^OE^ cells, especially after LPS stimulation, which inhibited the activation of ASK1. This indicated that the activity of Trx may be closely linked to the ASK1 pathway. Based on our current study, we propose a possible model of cellular signaling and apoptotic cell death regulation by bifunctional Prx1, which is shown in Fig. 7. Under physiological conditions, Prx1 overexpression stimulates the increased expression of ASK1 and triggers its downstream cascades such as JNK and p38. However, under pathological conditions such as LPS stimulation, the peroxidase of Prx1 exerts a protective effect on cellular toxicity against increased cellular H_2_O_2_ levels induced by oxidative stress^23,32,33^.

In summary, we showed that LPS-induced apoptosis, which is critically mediated by Prx1 and abolished by NAC and NQDI, is dependent on the coordinated mechanisms of ROS and ASK1 signaling. However, the biological relevance of these putative cellular functions of the peroxidase of Prx1 is currently unclear. Moreover, its other functions of regulating Trx oxidation and the Trx–ASK1 interaction are also complicated by proinflammatory responses as well as LPS-induced apoptosis. Nevertheless, an understanding of the role of bifunctional Prx1 in association with ROS and ASK1 may suggest strategies to manipulate the apoptotic pathway induced by either oxidative stress or LPS, and could aid the development of a diagnostic biomarker for major diseases.

## Materials and methods

### Cell culture and reagents

MC3T3-E1 cells, an osteoblast-like cell line, was bought from the Shanghai Cell Center. *Escherichia coli* LPS (serotype 055:B5) and H2DCFDA were purchased from Sigma Chemical Co (St. Louis, MO, USA). NAC and NQDI-1 were purchased from MedChem Express (MCE, NJ, USA). Antibodies to Prx1, Trx, Bax, Bcl-2, JNK, P38, Cyto-c, p-JNK, P-P38, ASK1 and Caspase 3 were purchased from Abcam (Shanghai, China). Anti-GAPDH and anti-p-ASK1 were purchased from Proteintech (Wuhan, Hubei, China) and Affinity (OH, USA) respectively.

### Detection of cell proliferation and apoptosis

Cells were plated in 96-well culture plates at a density of 5 × 10^3^cells/ml. After 24 h of incubation, cells were treated with different concentrations of LPS for the indicated time periods. Cell viability was measured by the Cell Counting Kit-8 (CCK8, solarbio, Beijing, China) assay. In addition, cell apoptosis was evaluated by flow cytometry using Annexin V-FITC/PI Kit (Hanbio, Shanghai, China). Briefly, cells cultured in 12-well dishes were trypsinized and stained with PI-conjugated anti-Annexin V antibody under darkness for 20 min at room temperature, then analyzed by flow cytometry using Accuri C6 plus (Becton Dickinson, Franklin Lakes, NJ, USA).

### Quantitative real-time PCR analysis

Relative mRNA levels of Prx1 and GAPDH were investigated by qRT-PCR. The Prx1 primer pair consisted of forward primer 5′-TCCCACGGAGATCATTGCTT-3′ and reverse primer 5′-GTGCGCTTGGGATCTGATAC-3′. GAPDH was utilized as a loading control with forward primer 5′-TGGCCTTCCGTGTTCCTAC-3′ and reverse primer 5′-GAGTTGCTGTTGAAGTCGCA-3′. Real-time polymerase chain reaction amplifications labeled with SYBR Premix Ex Taq (Takara Bio Inc., Shiga, Japan) were performed in a Roche 480 LightCycler (Roche, Mannheim, Germany).

### Measurement of intracellular reactive oxygen species

Intracellular ROS generation was measured using H2DCFDA. After inducing, cells were washed by α-minimum essential medium without fetal bovine serum for three times, and incubated with 10 μM H2DCFDA at 37 °C for 20 min, then washed twice with phosphate-buffered saline (PBS), and centrifugated at 1500 rpm for 5 min. The cells were resuspended with PBS and analyzed by flow cytometry on a Accuri C6 plus instrument. (Becton Dickinson, Franklin Lakes, NJ, USA)

### Construction of MC3T3-E1 strains with a knockout and overexpressed PRX1 gene

PrxI gene knockout MC3T3-E1 cell line used in this study were produced in previous research by the CRISPR/Cas9 system^39^. The cells with highly expressed Prx1 gene were generated by the Integration Operating System. In brief, retrieving the Prx1 gene coding sequence from the NCBI (National Center for Biotechnology Information), the purified gene segments were double-digested with *Eco*RV and *Pac*I to get cohesive end of Prx1 gene, then subcloned into pG-No(H)-CHO-Rosa(mutant AvrII)-FRT-(L)-h-its1vector (Genloci Biotechnologies Inc., Nanjing, China) to generate overexpress vector by T4 ligase, and subsequently transformed into competent JM109 *E. coli* cells, and then plasmid extracted from the bacteria was confirmed by DNA sequence analysis. The overexpressed vector and targeting oligo were electrotransformed into MC3T3-E1 cell, and the positive clone was screened and confirmed by real-time PCR.

### Western blot analysis

MC3T3-E1 cells were harvested after corresponding treatment and lysed in RIPA lysis buffer (Beyotime, Beijing, China) containing protease inhibitors. Protein concentrations were determined using BCA protein assays (Beyotime, Beijing, China). A total of 30 μg of each sample were added and separated by sodium dodecyl sulfate–polyacrylamide gel electrophoresis, then transferred to polyvinylidene fluoride membrane. The primary antibodies were added in 1:1000–5000 dilution and incubated at 4 °C overnight. Horseradish peroxidase-conjugated swine anti-rabbit IgG (DaKo, Glostrup, Denmark) was added and incubated at room temperature for 1 h. Western blot images were captured using a FluorChem E System (ProteinSimple, Santa Clara, CA, USA).

### Cell immunofluorescence

After treatment, cells on coverslips were fixed with ice cold methanol for 10 min, block specimen in 1% BSA-PBST (PBS + 0.1% Tween 20) for 60 min. Cells were incubated with primary antibodies against Prx1 overnight at 4 °C. Then, cells were incubated with FITC-Goat Anti-Rabbit IgG secondary antibody (Proteintech, Wuhan, Hubei, China) for 1 h at room temperature in the dark. After washing with PBS for three times, cells were incubated with 4',6-diamidino-2-phenylindole (DAPI; Abcam Ltd, Shanghai, China) for 5 min. Images were captured by fluorescence microscopy (Olympus BX53, Tokyo, Japan).

### Statistical analysis

All in vitro experiments were performed at least three times. All data are presented as mean ± SD. Differences between two groups were performed using *t*-test. Differences among groups were tested by one-way analysis of variance (ANOVA). Multiple-comparison tests were applied only when a significant difference was determined by the ANOVA. *P* values < 0.05 were deemed to be statistically significant.
